# Early Palliative Care Following Aborted Cancer Surgery: Results of a Prospective Feasibility Trial

**DOI:** 10.1097/AS9.0000000000000520

**Published:** 2024-11-18

**Authors:** Jordan M. Cloyd, Rakhsha Khatri, Angela Sarna, Lena Stevens, Victor Heh, Mary Dillhoff, Alex Kim, Timothy M. Pawlik, Aslam Ejaz, Sharla Wells-Di Gregorio, Erin Scott, Sachin S. Kale

**Affiliations:** From the * Division of Surgical Oncology, Department of Surgery, The Ohio State University Wexner Medical Center, Columbus, OH; † Department of Surgery, UT Southwestern Medical Center, Dallas, TX; ‡ Division of Surgical Oncology, Department of Surgery, University of Illinois Chicago, Chicago, IL; § Division of Palliative Medicine, Department of Internal Medicine, The Ohio State University Wexner Medical Center, Columbus, OH.

**Keywords:** clinical trial, gastrointestinal cancer, hepatopancreatobiliary cancer, quality of life, supportive care

## Abstract

**Background:**

Although resection is generally necessary for curative-intent treatment of most solid organ cancers, surgery is occasionally aborted due to intraoperative findings. Following aborted cancer surgery, patients have unique care needs that specialized palliative care (PC) providers may be best equipped to manage. We hypothesized that early ambulatory PC referral following aborted cancer surgery would be feasible and acceptable.

**Methods:**

This single-institution prospective clinical trial enrolled adult patients with gastrointestinal or hepatopancreatobiliary cancer with no prior PC exposure who had curative-intent oncologic surgery that was unexpectedly aborted. The primary endpoint was the completion of an ambulatory PC consultation within 30 days of enrollment. Secondary outcomes included changes in standardized measures of quality-of-life (QOL) and anxiety/depression during the 3-month follow-up.

**Results:**

Among 25 enrolled participants, the mean age was 65.3 ± 9.9 years, 68% were male, and 88% were White. The most common types of cancers were pancreatic (44%), hepatobiliary (20%), and colorectal (12%); reasons for aborting surgery were occult metastatic disease (52%) and local unresectability (36%). Only 13 of 25 (52%) met the primary endpoint of ambulatory PC within 30 days, less than the prespecified threshold of 70%. Overall, 16 (64%) patients completed ambulatory PC consultation a mean of 29.2 ± 15.8 days after enrollment. Of the 9 (36%) who did not, reasons included patient preference (n = 4), withdrawal from study (n = 1), lost to follow-up (n = 1), scheduling conflict (n = 1), and required inpatient PC before discharge (n = 2). Anxiety (4.94 ± 3.56 vs 3.35 ± 2.60, *P* = 0.06), depression (4.18 ± 4.02 vs 4.76 ± 3.44, *P* = 0.49), and QOL (82.44 ± 11.41 vs 82.03 ± 15.37, *P* = 0.92) scores did not significantly differ at 3-month follow-up compared to baseline.

**Conclusions:**

Barriers to early ambulatory palliative care consultation exist after aborted cancer surgery. Given the unique and complex care needs of this patient population, additional research is needed to optimize supportive care strategies.

## INTRODUCTION

Surgical resection is a critical component of the multidisciplinary, curative-intent treatment for most solid organ cancers. Unfortunately, even with a meticulous preoperative evaluation of patients’ physical status, individual cancer characteristics, and anatomic extent of the tumor, the intended procedure may be unexpectedly aborted, which is most often due to either occult metastatic disease or tumor unresectability.^1,2^ Rates of aborted cancer surgery (ACS) vary substantially based on the cancer type, specific patient characteristics, and selection criteria used to make decisions about surgery but may be as high as 25% in some high-risk scenarios.^3–7^ For most cancer types an aborted surgery is typically associated with a worse prognosis and inability for curative outcome.

In addition to the physical demands of postoperative recovery and risks of complications, patients with ACS also experience psychosocial distress resulting from a sudden change in prognosis, uncertainty about the next treatment course, and any residual side effects of the tumor left in situ.^8,9^ In fact, a recent qualitative study noted the impact of ACS on multiple aspects of health-related quality-of-life (HrQOL).^9^ Along with postoperative symptoms, patients reported a wide range of emotional symptoms, disruptions to normal life routines, and relied on a wide range of coping strategies while recovering.^9^ At the same time, a recent national survey of surgical oncologists found that while surgeons reported feeling comfortable addressing the surgical and cancer-related needs of their patients after ACS, they were less comfortable assessing and addressing psychosocial and symptom-control needs.^10^

Taken together, these findings suggest the potential need for specialized providers to help address the unique and complex care needs of this patient population. Palliative care (PC), with its focus on patient preferences, prioritizing symptom management, addressing psychosocial needs, and assisting with decision-making may enhance a patient-centered, multidisciplinary approach to patient care after ACS. Although traditionally reserved for patients with advanced disease, previous research has suggested that late referrals to PC are suboptimal for addressing patient needs.^11,12^ In fact, a large body of evidence highlights the clinical benefits of early PC referral among patients with newly diagnosed advanced cancer.^13–17^ Despite the empiric benefits of early PC integration with standard oncologic care and its endorsement by oncology societies,^18,19^ numerous institutional, provider, and patient-level barriers to its timely utilization exist.^20–25^ Therefore, the purpose of this prospective pilot trial was to determine the feasibility, acceptability, and preliminary efficacy of early ambulatory PC following ACS.

## METHODS

### Study Design and Population

A single-institution, single-arm prospective clinical trial was conducted between February 2022 and April 2024 at a major quaternary cancer center. Patients with gastrointestinal or hepatopancreatobiliary cancer who underwent aborted curative-intent oncologic surgery were prospectively identified and offered enrollment in a pilot trial of early ambulatory PC referral. Participants were eligible if they were aged ≥18 years, within 1 month of ACS, and had no prior PC consultation. Potential participants were identified by their surgical oncologists and referred to the research team for screening. Once eligibility was verified, patients were approached by a research coordinator either in the hospital or at a previously scheduled outpatient appointment. The coordinator described the role of PC in general terms, explained the specifics of the clinical trial, and provided a brief educational pamphlet with information about PC and details of their scheduled appointment.

For individuals wishing to participate, written consent was obtained, and the patient completed the baseline patient-reported measures. A referral to a board-certified PC physician was placed, and an ambulatory appointment was scheduled as soon as feasible. PC appointments were aligned with their regularly scheduled surgical or medical oncology appointments whenever possible to minimize travel demands. Subsequent visits with the PC team occurred as needed. Guidelines for the PC consultation were adapted from the National Consensus Project for Quality PC but included an emphasis on assessing and treating physical and psychosocial symptoms, assisting with decision-making, establishing goals of care as appropriate, and coordination among care providers.^26^ The study team contacted the subject 3 months after enrollment to complete the follow-up measures. All data were stored using REDCap until it was exported for analyses. The study was approved by The Ohio State University Wexner Medical Center Institutional Review Board (Protocol #2021C0156). Participants also received small financial compensation for their participation in the study.

### Primary Outcome

The primary outcome of the trial was the feasibility of early PC referral following ACS, which was defined as the proportion of enrolled patients who completed the initial ambulatory PC consultation within 30 days of enrollment. This trial was designed to assess whether the intervention was feasible for ≥70% of participants, which was similar to targets of prior pilot trials of PC referral.^14^ A sample size of 25 patients was deemed necessary to demonstrate a 70% feasibility rate with 90% confidence interval and a margin of error of 0.15.

### Secondary Outcomes

The secondary outcomes included changes in HRQoL, anxiety/depression, and prognostic awareness 3 months after enrollment. Mood was assessed using the 14-item Hospital Anxiety and Depression Scale (HADS).^27^ The HADS consists of 2 subscales assessing anxiety and depression symptoms in the past week, with subscale scores ranging from 0 (no distress) to 21 (maximum distress). HRQoL was assessed using the Functional Assessment of Cancer Therapy–Hepatobiliary Questionnaire v.4 (FACT-Hep).^28^ The FACT-Hep contains 45 items that comprise 5 subscales assessing physical well-being (PWB), functional well-being (FWB), emotional well-being, social well-being, and hepatobiliary cancer subscale specific concerns during the past week. We calculated the following summations: Trial Outcome Index (PWB, FWB, hepatobiliary cancer subscale), general quality-of-life (PWB, FWB, emotional well-being, social well-being), and cancer-specific HRQoL (all subscales). Higher total and subscale scores indicate better HRQoL. Finally, participants also reported information preferences and prognostic understanding using the 10-item Prognosis and Treatment Perception Questionnaire (PTPQ).^29,30^ The PTPQ assesses patient beliefs regarding the likelihood of cure, the importance of knowing about prognosis, and preferences for information about treatment; no scoring rubric is available.

Health care utilization, defined as the number of PC visits, number of emergency room visits, and hospital admissions, as well as opioid utilization, advanced care planning documentation, and oncologic treatment information were also collected during the 3-month follow-up. At the study endpoint, participants were sent a 5-question debriefing survey that focused on the utility and acceptability of the PC referral, which was assessed via the prompt “Did you find the referral to PC useful?” Additionally, a research team member conducted cognitive debriefing interviews with a small patient sample to gauge in-depth patient perceptions on the feasibility and acceptability of early PC referral. All interviews were conducted over the phone, audio recorded, and then transcribed by research personnel.

### Statistical Analysis

Standardized scoring systems were used to quantify patient responses on HADS, FACT-Hep, and PTPQ. Descriptive statistics for continuous and categorical variables were presented as mean (standard deviation) and frequency (%), respectively. Differences between overall and subscale measures at baseline and 3-month follow-up were assessed using linear mixed models accounting for within-subject correlations and individual differences. Statistical significance was defined at *P* < 0.05. The effect sizes in between groups comparisons were reported based on Cohen’s d effect size (small effect = 0.2–0.49, medium effect = 0.5–0.79, large effect = 0.8–1.19).^31,32^ Provider-reported content of the PC consultations was assessed by calculating the frequency of each theme/domain discussed. Patient interviews were coded by trained researchers and analyzed for consistent themes by trained research personnel. All statistical analyses were conducted using SPSS version 28 (IBM Corporation, Armonk, NY).

## RESULTS

### Patient Characteristics

**FIGURE 1.**
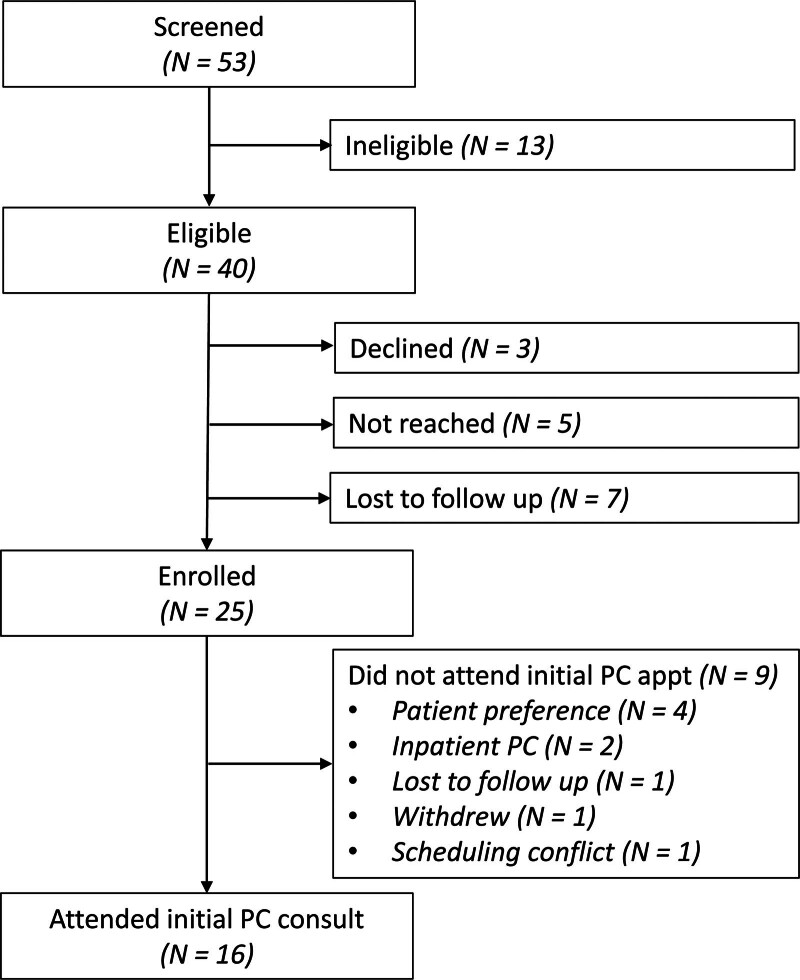
Study recruitment for early palliative care after aborted cancer surgery

Between January 2022 and December 2023, 53 patients who experienced ACS were screened for the trial. Among 40 potentially eligible patients, 25 were successfully enrolled, 7 were lost to follow-up, 5 were unable to be reached, and 3 declined participation (Fig. 1). Among the included patients, mean age was 65.3 ± 9.9 years (range 44–83 years); most patients were male (68%), White (88%), and married (75%). Cancer types included pancreatic (44%), hepatobiliary (20%), and colorectal (12%). The most common aborted operations included pancreatoduodenectomy (44%), hepatectomy, (36%), and peritoneal resection (12%). The most common reasons for aborting surgery included occult metastatic disease (52%) and local tumor unresectability (36%). Complete participant characteristics are reported in Table 1.

**TABLE 1.** Patient Clinical and Demographic Characteristics (n = 25)

Patient Characteristics	n (%)
Age	
Years ± SD	65.3 ± 9.9
Gender	
Female	8 (32)
Male	17 (68)
Race	
White	22 (88)
Asian	1 (4)
Not reported	2 (8)
Ethnicity	
Non-Hispanic/Latino	24 (96)
Hispanic/Latino	1 (4)
Cancer type	
Pancreatic	11 (44)
Hepatobiliary	5 (20)
Colorectal	3 (12)
Other	6 (24)
Aborted procedure	
Pancreatoduodenectomy	11 (44)
Hepatectomy	9 (36)
Peritoneal resection	3 (12)
Distal pancreatectomy	1 (4)
Reason for aborted surgery	
Occult metastatic disease	13 (52)
Local unresectability	9 (36)
Intraoperative risk assessment	3 (12)
Cancer treatment after surgery	
Chemotherapy	17 (68)
Radiation	5 (20)
Surgery	1 (4)
Other	7 (28)
Vital status	
Alive	24 (96)
Deceased	1 (4)
Distance traveled for PC	
Miles ± SD	70.7 ± 46.6
Time between ACS and first PC visit (days)	
Days ± SD	29.2 ± 15.8

Among the 25 enrolled patients, only 13 (52%) met the primary endpoint of ambulatory PC referral within 30 days of enrollment. Overall, 16 (64%) completed an ambulatory PC consultation a mean of 29.2 ± 15.8 days after enrollment. Of the 9 patients (36%) who did not attend an ambulatory PC visit, reasons included patient preference (n = 4), withdrawal from the study (n = 1), lost to follow-up (n = 1), scheduling conflict (n = 1), and required inpatient PC before discharge (n = 2). Patients traveled a mean of 70.7 (± 46.6) miles for the PC visit. In exploring “patient preferences” as a reason for not attending PC consultation, the primary reasons were unclear need for the appointment and the desire to avoid further medical appointments and the associated travel.

### Quality-of-Life and Mood

Of the 25 enrolled participants, all completed the baseline assessments and 17 completed the 3-month follow-up assessments. Only 12 of these 17 participants attended ambulatory PC at least once. There were no significant differences in anxiety (4.94 ± 3.56 vs 3.35 ± 2.60, *P* = 0.06), depression (4.18 ± 4.02 vs 4.76 ± 3.44, *P* = 0.49), or global HrQOL (82.44 ± 11.41 vs 82.03 ± 15.37, *P* = 0.92) at 3-month follow-up compared with baseline (Table 2). Despite the lack of statistical significance, the observed medium effect size (Cohen d = 0.5) suggested a moderate difference in anxiety levels between baseline and 3-month follow-up. In subset analyses stratified by actual receipt of ambulatory PC within 30 days, anxiety was improved at 3 months compared to baseline among those who received PC (*P* < 0.05) but not in those who did not have PC consultation (*P* > 0.05); no differences in depression or HrQOL were identified.

**TABLE 2.** Quality-of Life, Anxiety, and Depression at Baseline and 3-Month Follow-Up

	Baseline	Follow-Up	Mean Difference	*P*	Cohen’s d (95% CI)
HADS anxiety	4.94 (3.56)	3.35 (2.60)	1.59 (3.18)	0.06	0.5 (−0.1 to 1.0)
HADS depression	4.18 (4.02)	4.76 (3.44)	−0.59 (3.43)	0.49	−0.17 (−0.65 to 0.31)
FACT PWB	22.21 (3.40)	21.99 (3.85)	0.22 (4.95)	0.86	0.05 (−0.43 to 0.52)
FACT SWB	24.06 (4.53)	23.49 (3.87)	0.57 (5.58)	0.68	0.1 (−0.38 to 0.58)
FACT EWB	18.56 (3.37)	19.24 (3.53)	−0.68 (3.11)	0.38	−0.22 (−0.70 to 0.27)
FACT FWB	17.64 (6.36)	17.32 (6.74)	0.32 (7.35)	0.86	0.04 (−0.43 to 0.52)
FACT-Hep HCS	55.1 (8.72)	53.73 (10.67)	1.38 (11.39)	0.63	0.12 (−0.36 to 0.60)
FACT-Hep TOI	94.95 (15.86)	93.04 (18.70)	1.92 (20.50)	0.71	0.09 (−0.38 to 0.57)
FACT-G	82.44 (11.41)	82.03 (15.37)	0.40 (15.34)	0.92	0.03 (−0.45 to 0.50)
FACT-Hep	137.57 (18.61)	135.76 (23.15)	1.81 (24.09)	0.76	0.08 (−0.40 to 0.55)

TOI, trial outcome index.

### Prognostic Awareness

Twenty patients completed the PTPQ at baseline while 17 completed it at the 3-month follow-up (Supplemental File 1, http://links.lww.com/AOSO/A433). At the end of the study period, most patients (81.3%) desired as much information as possible about their diagnosis and treatment and reported (76.5%) that it was extremely important to know the likely outcome of their cancer over time. Yet, the majority (64.7%) still reported the primary goal of their current cancer treatment was “to cure my cancer” and 50% reported that it was either extremely or moderately likely that they “will be cured of cancer.” The majority (82.4%) of patients stated that they had not discussed end-of-life care with their treating oncologist and yet few (11.8%) wished they “had more information about their prognosis.”

### Palliative Care and Healthcare Utilization

During the 3-month study period, patients attended a mean 1.67 (range: 1–3) PC visits. During PC visits, providers spent the greatest percentage of time in the domain of symptom management. The most addressed symptoms included pain (66.7%), constipation (40%), nausea (30%), fatigue (26.7%), insomnia (16.7%), and depression/anxiety (10%). As such, PC physicians provide a range of interventions, including physical, psychosocial, behavioral, spiritual, and end-of-life planning. The most common interventions were therapeutic listening (70%), utilization of supportive statements (66.7%), pharmacotherapy management for pain (53.3%), meaning-focused exploration of illness and treatments (53.3%), and exploration of hopes and worries (46.7%). During the 3-month enrollment period, patients had an average 0.64 (range: 0–4) ER visits and 0.59 (range: 0–2) hospital admissions. One patient who did not have an ambulatory PC visit (but did see inpatient PC) died during the study period.

### Utility and Acceptability

Of the 17 patients who completed the 3-month follow-up survey, 11 reported the PC referral as useful whereas 5 responded it was not and 1 did not answer. Representative examples of reasons why PC was useful included “understanding I have support,” “gave me ideas about what to expect in the future,” “pain care,” “talking about my cancer,” and “second set of ears and eyes to discuss things.” After study completion, 5 patients participated in a cognitive debriefing interview. Overall, 4 participants’ experience was generally positive despite initial reservations stemming from the unfamiliarity with PC. The availability of specialized providers to focus on symptom management was rated highly by participants. As one stated, rather than, “bringing more excess stuff to my [oncologists], I can have someone [else] paying attention to that aspect of my care.” The 1 participant who voiced an unfavorable opinion of PC referral felt that their symptoms were still poorly controlled despite the additional appointment. In addition to patient benefits, many participants perceived PC referral as helpful for their caregivers. The primary barrier to PC referral after ACS was transportation and scheduling.

## DISCUSSION

Unfortunately, despite the use of contemporary diagnostic tools to aid careful patient selection and evaluation, ACS still occurs relatively commonly even at high-volume experienced cancer centers.^1^ Following ACS, these patients experience many of the same surgical symptoms, need for recovery, and risk of complications as patients who undergo successful curative-intent surgery, while simultaneously experiencing the psychosocial distress of a terminal change in their prognosis, as well as the side effects of the cancer left in situ.^8^ Recent qualitative research has highlighted the significant impact of ACS on patients’ physical and emotional well-being and suggested the need for specialized providers focused on managing patient symptoms, facilitating decision-making, and coordinating oncologic care.^9^ Based on the existing evidence of improved outcomes with early PC among patients with advanced malignancy, the investigators hypothesized that incorporating PC after ACS might improve patient outcomes and therefore sought to test the feasibility and acceptability of ambulatory PC referral after ACS.

The results of the current prospective pilot trial suggested that significant barriers exist to early ambulatory PC after ACS, which may not be surprising given the existing literature on PC integration with oncologic care. Indeed, despite its empiric benefits and endorsement by oncology societies, numerous barriers to its utilization in patients with advanced cancer have been well documented.^18,19,33,34^ For example, lack of knowledge about and misperceptions regarding PC are common among patients and can lead to reluctance to accept PC referral.^35–39^ In addition, an inadequate workforce of specialized PC providers along with other institutional and provider-level barriers may limit the timely scheduling of patients with serious illness.^20–25^ Specifically for patients who have experienced ACS, the same factors that increase the need for PC services (ie, symptoms from cancer and recent surgery, need to schedule/coordinate multiple other provider visits, psychosocial distress from recent diagnosis) may present challenges to timely PC consultation. Even within the structure of a prospective clinical trial (eg, research coordinators, priority scheduling, etc.), similar barriers were observed in our study, and these should be the focus of future research.

Several secondary outcomes were assessed to evaluate the preliminary efficacy of PC referral following ACS. While these analyses were limited in that follow-up data were available for only 17 patients, 30% who did not attend PC consultation, no significant differences were observed in HrQOL or anxiety/depression on the HADS scale. This finding is in contrast to prior literature demonstrating that, among patients with advanced cancer, integration of PC with standard oncologic care improves QOL, facilitates better communication, enhances patient and caregiver coping skills, and reduces healthcare utilization at the end of life.^14,16,40^ While the study was not powered to detect differences in these secondary endpoints, subset analyses did suggest an improvement in anxiety levels among those patients who did attend ambulatory PC consultation but not among those who did not attend PC. Interestingly, follow-up assessments found little improvement in patient understanding of their prognosis despite previous research noting improved prognostic awareness following PC involvement.^41^ This finding may be related to the observation that discussions regarding prognosis and end-of-life care were relatively uncommon during the initial PC visits.

While the benefits of early PC integration are well established in patients with advanced cancer treated by medical oncology,^42^ the role of PC in surgical oncology remains currently undefined.^43^ While PC integration into surgical oncology clinics has been shown to improve advanced care planning,^44^ an important quality metric,^45^ the impact of specialty PC on surgical outcomes has until recently been unclear. Two recent randomized controlled trials investigated the effect of routine perioperative specialty PC consultation on the outcomes of patients undergoing elective major intra-abdominal cancer surgery, with neither demonstrating improved HrQOL nor clinical outcomes at 3 months postoperative versus usual care.^46,47^ These findings suggest that specialty PC may be less helpful in patients with early-stage cancers who are physically and emotionally optimized for curative-intent surgery and may be more beneficial in targeted high-risk populations with complex care needs. Yefimova et al^48^ utilized data from the Veterans Affairs Healthcare System and reported an association between receipt of perioperative PC consultation and improved caregiver ratings of end-of-life care, communication, and support among patients who died within 90 days of high-risk surgery. In this study, the majority of PC consultations were performed postoperatively and as inpatient. Future work in patients who experience ACS may consider earlier supportive care strategies, perhaps while as an inpatient, to address early patient distress and overcome some of the barriers observed in our trial. Alternative approaches could include the utilization of telehealth technology, training surgeons to provide primary PC, and/or individualized assessment through routine screening of patients’ supportive care needs so that specialty PC can be delivered to those at the highest risk for distress.

While the results of this prospective feasibility study are highly relevant for defining the role of specialty PC after ACS, several limitations should be acknowledged. First, this was a single-institution trial at a major Midwestern academic medical center and thus the findings may not be generalizable to other health care settings. Second, while PC providers were experienced and followed best practices for delivering PC, the PC consultations were otherwise not standardized. Third, the sample size was relatively small and limited by incomplete 3-month follow-up assessments. On the other hand, the trial was designed and powered for feasibility; data from secondary endpoints will be helpful to design future trials in this patient population.

In conclusion, among patients undergoing planned curative-intent resection who experience ACS, significant barriers to early ambulatory PC consultation were observed. Given the unique and complex care needs of this patient population, additional research is needed to optimize supportive care strategies following unsuccessful cancer surgery.

## Supplementary Material
