# Supplementary material for: Early Palliative Care Following Aborted Cancer Surgery: Results of a Prospective Feasibility Trial
Source: Ann Surg Open. 2024 Nov 18;5(4):e520. doi: 10.1097/AS9.0000000000000520 (PMC11661761; doi:10.1097/AS9.0000000000000520)
Supplement: Supplementary file 1 [file as9-5-e520-s001.pdf]

Variable	Level	Baseline<br>N=20	Followup<br>N=17
Q1:Preference for details of information about diagnosis and treatment	I prefer not to hear a lot of details.	1 (5.0%)	0 (0.00%)
	I want to hear as many details as possible in all situations relating to my cancer and its treatment	14 (70.0%)	13 (81.3%)
	I want to hear details only in certain situations, such as when tests are abnormal or when treatment	5 (25.0%)	3 (18.8%)
Q2:Rate the quality of the information given	Excellent	13 (68.4%)	12 (70.6%)
	Good	4 (21.1%)	4 (23.5%)
	Satisfactory	2 (10.5%)	1 (5.9%)
Q3:Primary goal of current cancer treatment	For me and/or my family to be able to keep	0 (0.00%)	2 (11.8%)
	To cure my cancer	11 (55.0%)	11 (64.7%)
	To extend my life as long as possible	4 (20.0%)	3 (17.6%)
	To help cancer research	1 (5.0%)	0 (0.00%)
	To lessen my suffering as much as possible	1 (5.0%)	0 (0.00%)
	To make sure I have done everything	3 (15.0%)	1 (5.9%)
Q4:Oncologist's primary goal of current cancer treatment	For me and/or my family to be able to keep	0 (0.00%)	3 (17.6%)
	To cure my cancer	12 (60.0%)	7 (41.2%)
	To extend my life as long as possible	5 (25.0%)	5 (29.4%)
	To lessen my suffering as much as possible	2 (10.0%)	1 (5.9%)
	To make sure I have done everything	1 (5.0%)	1 (5.9%)
Q5:Importance to know the likely outcome of cancer over time	A little important	1 (5.0%)	0 (0.00%)
	Extremely important	14 (70.0%)	13 (76.5%)
	Somewhat important	0 (0.00%)	1 (5.9%)
	Very important	5 (25.0%)	3 (17.6%)
Q6:Frequency of conversation with oncologist about outcome of cancer over time	Often	11 (55.0%)	6 (37.5%)
	Rarely	2 (10.0%)	2 (12.5%)
	Sometimes	6 (30.0%)	4 (25.0%)
	Very Often	1 (5.0%)	4 (25.0%)
Q7:Feelings about amount of information of outcome of cancer over time	I now have about the right amount of information.	13 (68.4%)	15 (88.2%)
	I wish I had more information about my prognosis.	6 (31.6%)	2 (11.8%)
Q8:Quality of information by oncologist about outcome of cancer over time	Excellent	7 (35.0%)	8 (47.1%)
	Good	11 (55.0%)	7 (41.2%)
	Satisfactory	2 (10.0%)	2 (11.8%)
Q9_1:Making decisions about treatment	Extremely helpful	10 (52.6%)	12 (70.6%)
	Very Helpful	9 (47.4%)	5 (29.4%)
Q9_2:Preparing for the future	A little helpful	3 (15.8%)	2 (12.5%)
	Extremely helpful	9 (47.4%)	11 (68.8%)
	Very Helpful	7 (36.8%)	3 (18.8%)
Q9_3:Maintaining hope	A little helpful	2 (10.5%)	3 (17.6%)
	Extremely helpful	9 (47.4%)	10 (58.8%)
	Very Helpful	8 (42.1%)	4 (23.5%)
Q9_4:Coping with the disease	A little helpful	2 (10.5%)	2 (11.8%)
	Extremely helpful	10 (52.6%)	10 (58.8%)
	Very Helpful	7 (36.8%)	5 (29.4%)
Q9_5:How helpful knowledge is about prognosis	A little helpful	2 (10.5%)	2 (12.5%)
	Extremely helpful	10 (52.6%)	10 (62.5%)
	Very Helpful	7 (36.8%)	4 (25.0%)
Q10:Describe current medical status?	Relatively healthy	13 (68.4%)	9 (52.9%)
	Relatively healthy and terminally ill	4 (21.1%)	7 (41.2%)
	Seriously ill and not terminally ill	1 (5.3%)	0 (0.00%)
	Seriously ill and terminally ill	1 (5.3%)	1 (5.9%)
Q11:How likely do you think it is that you will be cured of cancer?	Extremely likely (more than a 90% chance of cure)	7 (35.0%)	4 (25.0%)
	Moderately likely (50-74% chance of cure)	5 (25.0%)	4 (25.0%)
	No chance (0% chance of cure)	2 (10.0%)	1 (6.3%)
	Somewhat likely (25-49% chance of cure)	0 (0.00%)	1 (6.3%)
	Unlikely (10-24% chance of cure)	1 (5.0%)	1 (6.3%)
	Very likely (75-90% chance of cure)	3 (15.0%)	2 (12.5%)
	Very unlikely (less than 10% chance of cure)	2 (10.0%)	3 (18.8%)
Q12:Discussed wishes about care to receive if dying	No	20 (100.0%)	14 (82.4%)
	Ye	0 (0.00%)	3 (17.6%)
Q13: Preference for exdeding life or relief of pain	Don't know	7 (35.0%)	7 (41.2%)
	Extend life as much as possible	9 (45.0%)	8 (47.1%)
	Relieve pain as much as possible	4 (20.0%)	2 (11.8%)
